# Selections and Abstracts

**Published:** 1902-01

**Authors:** 


### SELECTIONS AND ABSTRACTS.

---

### HYDROPHOBIA, WITH REPORT OF THREE CASES*

Of the three cases which I wish to report to-night, two occurred under my own observation in 1899, and the third is of recent occurrence in the practice of Dr. Thomas Hubbard, of Toledo, who kindly allows me to report it with my own.

The first patient, Mr. L., 71 years of age, was seen at the request of Drs. J. F. and W. S. Hobson, on May 30, 1899. Until the development of this difficulty he was in good health. During the very cold weather early in February he went to the barn to milk, and was bitten on the hand by a large tomcat, which sprang upon him without obvious reason. He had been in the habit of petting the cat, which seemed to be fond of him. He allowed about one-half hour to elapse, and the wound was then cauterized and treated with an antiseptic dressing. The cat had been observed to be sick

---

*Read by Henry S. Upson, M.D., Professor of Diseases of the Nervous System in the Western Reserve Medical School, Cleveland, before the Cleveland Medical Society, October 11, 1901.

for two or three days previous to this time, was very irritable, wild, and apparently delirious. About three or four days after the bite was inflicted, the cat died. The body was frozen stiff. A rabbit was inoculated from this cat's spinal cord by Dr. Perkins, and at the time when the patient was first seen, some four months after the bite, the rabbit, and a cat which had been bitten by the presumably rabid one, were both under observation in the laboratory of the Lakeside Hospital. They were living and well.

The patient was quite well until May 25, when pains began to shoot up from the hand along the arm. This continued for two days. On the 28th the patient was restless, but had a fairly good night's sleep after taking two teaspoonfuls of bromidia, and 1.50 grain of hyoscyamin sulphate. When seen by me on the morning of May 30 he had developed considerable difficulty in swallowing. This had been noticed for the first time the night before. It was especially hard for him to swallow water or other fluids. He could not eat any food, although the night before he had eaten a few strawberries and that morning he had eaten two strawberries. He found, however, not much difficulty in taking pills or capsules. In attempting to swallow water he manifested great distress, breathed very fast, sighed deeply, and usually gave it up, although he did manage at times to swallow some fluid. His mind was perfectly clear, he talked rationally, not only on the subject of his illness, but on other subjects, and did not seem very much agitated in view of the circumstances, but was quite sure that he had hydrophobia. The pulse was 100, and the temperature previously taken was also 100°. The pupils were of normal size and reacted well to light. The knee-jerks were both fairly lively, the one on the left side rather the more so. The arteries were considerably sclerosed, and a blowing systolic murmur was heard all over the precordium, most marked over the base of the heart. When seen again on the same day, at 10:30 p.m., the difficulty in swallowing had markedly increased. The patient was very restless, and attacks of distress and rapid breathing came on frequently without any special cause. The patient still talked rationally, but had eaten nothing during the day, and had evidently grown weaker. A teaspoonful and a half of the bromidia was given with two ounces of milk by the rectum at 11 p.m. At 12:30 a.m., the patient was not perceptibly better, and was given one-quarter of a grain of morphin by hypodermic. During the night he slept a few minutes at a time, and then would wake in a paroxysm, would jump out of bed and would sometimes insist on going out into the open air. During the night he had grown more and more confused. On the morning of the 31st, he recognized his relatives and friends, but wandered from one subject to another and was decidedly delirious. He staggered markedly when he walked, and had apparently lost in flesh. His pulse was 120. During the night he succeeded in taking a half glass of water by the mouth. After the administration of the bromidia and the morphin, the patient insisted on washing his face and made several attempts to drink water, but without avail. When he washed himself he had fairly severe spasms of the throat muscles, but not more intense than those of the day before.

At four o'clock in the afternoon of the same day the patient was weaker and more delirious. He was in bed, and did not offer to leave it. The temperature had risen to 103° and the pulse was 120. The speech, which, when he was first seen on Tuesday morning showing slight blurring, had become more and more indistinct. This condition deepened during the night, and the patient passed gradually into a condition of muttering delirium bordering on coma. In the morning he was for a time unconscious, and the movements of the hands, legs and face reduced themselves to mere twitchings. The patient at this time was under the influence of morphin. As this passed off he became somewhat more restless and was given another injection. The patient became progressively weaker, and death occurred at 1:30 p.m. Fairly marked tympanites developed during the night, and 12 or 13 ounces of urine were drawn with the catheter. The urine was of a dark amber color and clear. I regret that it was not examined chemically, but with the excitement consequent on catheterization, which was carried on with great difficulty, it was thrown away. During the night there began to be stiffness of the neck-muscles with a tendency to opisthotonos, and the muscles of the back were also quite stiff. It distressed the patient a great deal to be moved. No paralysis developed in any part of the body. The pupils remained equal and reacted to light throughout the illness.

Two weeks after the patient's death, the rabbit which had been inoculated from the cat's spinal cord died with typical symptoms of hydrophobia of the paralytic variety. Another rabbit was inoculated from the spinal cord of this one, but both this animal and the cat, previously bitten, remained well.

I cannot help regarding this as a typical case of hydrophobia. The patient was an unusually well-balanced man with no hysteric tendency. While he somewhat feared hydrophobia and was convinced from the very first appearance of his illness that that was what he had, his conclusion was a perfectly rational one well borne out by the facts, and he carried himself throughout with wonderful nerve. With the appearance of such symptoms as he presented only two diseases were possible, hydrophobia and hysteria. The fact that hysteria sometimes simulates hydrophobia is not very significant in view of the fact that it imitates almost every other known disease. I do not myself believe it possible that hysteria can produce an ailment which runs a steadily progressive course and ends fatally within four days. The occurrence of muttering delirium passing into coma, not at all of a hysteric type, but much as it is seen in typhoid and other infectious diseases; the elevation of temperature; the stiffness of the muscles of the back and of the neck, with tenderness such as is sometimes seen in meningitis are not, in my opinion, capable of being simulated consciously or hysterically.

The second patient, Mrs. H., 45 years of age, was seen on Saturday, July 8, 1899. For the previous two weeks she had not been feeling well, and on Thursday, July 6, she first noticed that she had some difficulty in swallowing fluids. This developed very rapidly, so that by Friday morning she could swallow neither liquid nor food. When she attempted to do so, she had spasms of the throat muscles, evinced great distress, and had sighing respiration. She apparently had no idea what was the matter with her, but careful inquiry of her husband at this time developed the fact that two or three months before, in crossing the yard at night, a large black dog had put his front paws on her chest and struck the septum just inside the nares, either with his teeth or with a claw; presumably, from the subsequent illness, with his teeth. There was slight bleeding, but the patient and her friends were not at all alarmed, and paid no attention to the matter. It was practically forgotten. When the patient was seen on Saturday morning, July 8, 1899, she was found to be a fleshy woman of good color. She was sitting in a chair, was spitting thick, tenacious saliva very vigorously in all directions, and was in great distress. This distress was much increased by attempts at drinking, which set up typical spasms of the throat-muscles. At times she would start from her chair, and once or twice fell very heavily. The pulse was 120, soft and quite feeble, and the temperature 107°. The urine contained a moderate amount of albumen, hyalin casts, and a very perceptible amount of sugar. In spite of the fact that she had been given one-half grain of morphin by hypodermic, an hour afterward the pupils were widely dilated and reacted little, if at all, to light. In the evening of the same day she was found lying on the couch and tied with a sheet to prevent her from getting up. Her pulse was 140. She had ceased spitting, although she made a few attempts to do so. Her speech, which was somewhat thick when she was first seen, had become a muttering delirium, rather difficult to understand. She was perceptibly weaker. She went into coma, and died quietly at about ten o'clock the next morning. She only made one reference during her illness to the possibility of her having hydrophobia. This was on Friday evening, and she simply remarked that if she died she did not know what it could be unless it was hydrophobia. This idea was suggested to her apparently by her distress in attempting to swallow liquids, and she made no reference to her experience with the dog.

Physical examination of the thoracic and abdominal organs gave a negative result. The impression made on the observer by the general appearance and demeanor of this patient was not one of fear or of mental distress, but strongly suggested one suffering from an infectious disorder acting on the nerve-centers and the circulation.

The details of the third case are furnished to me by Dr. Thomas Hubbard, of Toledo. The patient was a young man 19 years of age, of excellent health and exemplary habits, employed as clerk in a grain-broker's office. On Thursday, August 29, 1901, he had coryza with some sore throat, for which he consulted his family physician. At that visit he talked with the doctor's wife for some time and to her surprise spoke about dying in a foreboding manner. He was usually very cheerful and hopeful and inclined to reticence. He worked at his desk for two days longer, but on Saturday, August 31, he seemed weak and exhausted. During this interval he had talked with the family about dying, and said that he had pains under his finger-nails and in the shoulder, with some sore throat. On Saturday morning he could not swallow milk, but ate some toast. There was no inflammation to account for the dysphagia. He had convulsions, apparently of hysteric character, at the mention of water, or if he heard it splashing in the room. There was a slightly frothy mucous in the throat which he spat in all directions when having a spasmodic attack. There were throat spasms, and at such times the face became purple. The physician in attendance said that the pulse was not feverish up to Monday evening. At midnight of Monday Dr. Hubbard found the patient with a temperature of 101.1° in the axilla, the pulse 160. The general appearance was that of a hysteric condition rather than that of one in morbid fear of death. The patient watched eagerly every movement, and when he spoke rationally it was only to exaggerate the symptoms mentioned in his presence. He answered questions for the most part rationally. Dr. Hubbard made a careful examination of the throat, larynx and trachea, but found no inflammation and no paralysis. He was given morphin and potassium bromid, and at about 4 o'clock on Tuesday morning he had a convulsion and told his father that he was going to die. The father left the room to call his wife, and the boy was dead when he returned. The body was embalmed within two hours after death, and no autopsy was permitted. No special tests were made to determine whether the death was real or apparent.

On inquiry it could not be learned that this patient had been bitten or in any way infected by a dog. About six months previous to his death, a pet dog belonging to his father was taken sick. The next day the dog went away and stayed all night, returned the next day very sick, and died on the following day without any violent symptoms whatever. The veterinary surgeon who saw him thought that he had been injured internally.

It seems to me that the diagnosis is in this case somewhat difficult. We may, I think, exclude a pseudohydrophobia from fear, as the boy himself never mentioned hydrophobia and his parents say it was not mentioned in his presence. The forebodings of death which he had were such as are found especially in diseases which affect the circulation, notably in angina pectoris. We are left to suppose either a general fear of death, which proved fatal, or the occurrence of some infection which ran a rapid and fatal course. As between hydrophobia and tetanus, the only two diseases suggested in such cases, it seems to me that tetanus may be with great probability excluded, as its symptoms are not present. That the pet dog which died six months ago may have had rabies, and that the rabies may have been in some way transmitted to the patient, is a possibility which it seems to me cannot in fairness be overlooked. Rabies of the paralytic form is common in dogs, and usually remains undiagnosed. Infection from such cases is rare, as the tendency to bite is absent, but sick dogs often lick the hand, and it should be remembered that, as Pope says:

> " Of all mad creatures, if the learned be right,
> It is the saliva kills and not the bite."

It may be well to add a few words on the diagnosis of the disease, in view of the fact that doubt is sometimes expressed by some physicians and many others whether such a disease as hydrophobia exists. Death is the great reality to both doctor and patient, and few diseases which run a progressive and fatal course have their genuineness called in question. Doubt is only made possible by two features of hydrophobia, the one its rarity, the other the fact that its serious manifestations are often obscured by nervousness, the outcome of terror on the part of the patient and others.

In distinguishing clinically between hydrophobia and its hysteric imitation I should be governed by the following considerations: that in the real disease the symptoms develop gradually; that delirum is not of sudden onset and early appearance but occurs some time after the appearance of the spasms in the throat; that together with the delirium come the signs of nerve exhaustion, the rapid pulse and often fever which usually accompany acute infectious diseases. In a well-marked case the clinical picture is as convincing as is that of typhoid fever. Death occurs in a way little, if at all, suggestive of exhaustion from fear, but seems to come as an overwhelming of the nerve-centers by a poison essentially depressive after a transitory stage of excitement. The hysteria is as often in the spectators as in the patient. It is easy to mistake chokings and rattlings in the throat for barking, and a calm view of so painful a disease is not to be expected from the friends and relatives. The delirium of the genuine cases should be distinguished from the hysteria of the spurious ones. Well-marked barking and attempts to bite the bystanders, while they might occur as hysteric additions to the symptom-complex in genuine cases, are strongly suggestive of malingering or of unconscious or hysteric imitation.

It is significant that of the three fatal cases here reported, in two of them the fear of hydrophobia was not present. With symptoms so well-marked the prognosis is quite hopeless, and the duty of the physician is to relieve pain and allay distress by adequate doses of morphin. Under the influence of this and other sedatives these patients need not suffer more than do patients dying from many other affections. An early diagnosis is doubly necessary in order that adequate sedatives may be given throughout the course of the disease.

DISCUSSION.

*Dr. W. T. Howard, Jr.:* It seems to me that it is hardly necessary in this late day to have to offer proof of the existence of such a well-established disease as rabies. It is, however, but fair to say that concerning one of Dr. Upson's cases there can be no question of the existence of true rabies. In this case the cat which bit the patient was brought to my laboratory at Lakeside Hospital and a rabbit inoculated from its medulla developed typical paralytic rabies. Cultures made from this rabbit after its death were sterile, thus excluding bacterial infection as the cause of the clinical symptoms and death.

*Dr. Frank E. Bunts:* I have been very much interested in this subject for a great many years, and have been very anxious to see a genuine case of rabies. I have been informed by those who have seen them that the symptoms are so characteristic that they cannot be mistaken for any other disease. It certainly is very rare in this part of the country, and yet the set of symptoms is so incomplete in many cases that we are pretty nearly right, I think, in deciding that there are not so many cases of it as we have thought. I think that a fear of water and spasm of the pharyngeal muscles might be properly called hydrophobia, but the exact cause of these symptoms has not been proved yet by the microscope or otherwise, and until the microorganisms can be found, we cannot be very positive what rabies is. In one case mentioned by Dr. Upson, there is one objection that might be made, and that is that the animal which gave the infection was a cat. Now, as I understand it, hydrophobia does not develop in cats except when bitten by a member of the canine family, as it is primarily a canine disease and cannot be communicated by a cat unless primarily bitten by a canine, and there has been no history given to prove that the cat was first bitten. And if it does not develop spontaneously in cats, we have no evidence to prove that it was a case of hydrophobia. Paralytic rabies was practically an unknown disease until the Pasteur treatment was introduced, and it has been suggested that it is a new disease caused by this method of treatment. The only thing we recognize as rabies in the human being is a more violent form characterized by spasms of the muscles of the larynx and pharynx. The paralytic form has been found in animals. There is one delusion that we have been laboring under, and that is that animals are afraid of water during these attacks. This is not true. On the contrary they want it, and try to drink it when they are unable to swallow, putting their heads into it but cannot drink though they try. The term "hydrophobia" arose from this delusion with regard to the fear of water, and rabies would be a much better term to call the disease. I am very anxious to see a case, but when I have tried to get at the facts of the cases reported in any of our local papers, the result has been that the physician would reply that there was nothing to report or that it has been overdrawn.

*Dr. A. R. Baker:* I do not wish to go on record as not believing there is any such thing as hydrophobia, but I do believe it a very rare disease. About twenty-five years ago Dr. Dulles of Philadelphia read a paper before the Pennsylvania State Medical Society, in which he expressed his doubt as to there being any such disease as hydrophobia. A committee on collective investigation was appointed to investigate the subject, and I was one of the committee, each member of which was to investigate and report upon every case in his own section of the State. I recall the fact that all of the cases I investigated were found to be cases of tetanus, hysteria or fright, and this appeared to be the experience of all the members of the committee. Since the establishment of the Pasteur Institutes, there have undoubtedly been numerous cases of the paralytic form of the disease reported; but during our earlier observation of the disease we probably should not have recognized these symptoms as hydrophobia. Since the establishment of the Pasteur Institutes there have undoubtedly been more deaths reported from rabies than formerly; whether the disease is on the increase, or whether the disease was not recognized, or whether the Institutions have disseminated the disease, I am unable to say, but in any event I believe we should discourage the yellow journals in their frequent outbursts of frenzy about mad dogs, as the disease is so rare that the chances are that most all of us will practice medicine a lifetime without ever seeing a case of rabies. So why the necessity of this great fear of a dog bite? We owe it to our patients to allay this morbid fear the same as we do that of ghosts and hobgoblins.

*Dr. J. F. Hobson, of Lakewood:* I wish to say that I was called in the first case reported by Dr. Upson, and I would like to correct one statement made in the paper, and that is in regard to the cauterization of the wound. The bite was given about 5 o'clock in the afternoon of one day, and I was not called until about 10 in the forenoon of the next day. I deemed it too late then for cauterization to be of any use, but thoroughly washed out the wound with a 1-1000 bichlorid solution. The cat had lived out in the pear orchard during the summer, and on the setting in of the fall rains and cold weather had come to the barn for winter quarters. During the fall and winter preceding the date of the bite in question, there was something radically wrong with the dogs and cats west of Rocky River. There were no cases of anything like rabies in the human subject, but there were many among brute animals. I do not quite agree with Dr. Baker that rabies is at least a very rare disease, and that many cases reported as rabies would, on investigation, prove to be meningitis or tetanus or some other infection, but on the contrary firmly believe that many cases reported as meningitis or tetanus would upon careful examination prove to be rabies. To the one who has seen a typical case of rabies, the diagnosis will not be difficult, as it appears entirely different from any other disease and is not to be mistaken for anything else. I wish to further state that I believe many cases of rabies in both the human and lower animals recover. The typical, classic cases always die.

*Dr. P. Maxwell Foshay:* I have never seen a case of rabies, and so have no personal knowledge of the subject under discussion. However, from some reading I have gathered one point which is of some importance in a discussion as to whether there is such a disease as hydrophobia. It is proved beyond all doubt that dogs suffer from a distinct disease which is called rabies. This disease is communicated from one dog to another by the bite of the infected animal. The disease spreads as an epidemic among dogs and other animals. It runs a uniformly fatal course, and has the characters of an infectious disease. The cerebrospinal fluid of an animal dead of the disease, when injected into a healthy animal, such as a rabbit, produces symptoms like those from which the infected animal suffered, and, if in sufficient dose, death. Possibly there may be cases of fatal hysteria in the human being, although I am not inclined to think so; but it certainly strains credulity to assume epidemics of fatal hysteria among dogs and other animals, the cases arising only after the bite of an animal already affected. Going a step farther we find that man contracts a disease with similar symptoms when he is bitten by animals suffering from this disease, and it is unreasonable to believe that this is always hysteria. Undoubtedly some so-called cases of hydrophobia are really hysteria, but that does not dispose of them all. A year or so ago Buffalo and Erie County, New York, suffered from an epidemic of rabies among dogs in which the source of infection was, as I recall it, carefully traced to a small Canadian town, and a study of this epidemic was published showing very clearly how the disease spread from one locality to another. It was not canine hysteria.

*The President:* I remember several years ago when some cases of hydrophobia were reported to this society. Some members were very skeptical as to the existence of rabies. Is it possible that the skeptics have been convinced of the existence of the disease?

*Dr. D. S. Hanson:* I saw a case which was reported to be hydrophobia, but I did not make that diagnosis. I did not know then that Dr. Bunts was so anxious to see all cases of so-called hydrophobia or I would have sent for him, and should have had some of the other doctors to see the case also. A boy had been shot in the foot and was suffering from tetanus. I advised the attending physicians to give carbolic acid hypodermically, and the boy made a good recovery. Meeting the doctor a few days after my visit, and inquiring about the boy, he said that the boy was better but had hydrophobia—that he was sure of it because the boy was barking like a dog. This is the statement almost always made, the choking in swallowing and the sounds made thereby being likened in the excited imagination to the barking of a dog. There is one thing which I think would aid in the diagnosis, and that is that we keep in mind the fact that the spasm in the throat which prevents swallowing is not the spasm of the pharyngeal muscles but of the larynx, and also that there is an absence of trismus in all cases, and death occurs on or before the end of five days.

*Dr. Henry S. Upson:* It is interesting to find that there is still a certain amount of skepticism in regard to the existence of hydrophobia. Doubts on this subject are not shared by writers of text-books on nervous diseases who agree in considering hydrophobia a disease as well marked and certain as are apoplexy and tabes. I cannot help agreeing with Gowers who thinks that doubt on the subject is only possible to one who has never seen a case of the disease. There are, as in other diseases, cases which are typical, those to which a certain amount of doubt may be attached, and spurious ones. Of the three cases which I have reported, the first one I must regard as established by a chain of evidence as complete as is possible. There was, in the first place, an outbreak of rabies among the dogs in the locality, transmitted to horses and cattle by biting. The cat by which the patient was bitten was typically rabid from a clinical point of view, and inoculation from his spinal cord produced typical rabies in a rabbit in the hands of the reliable observers connected with the laboratory of the Lakeside Hospital. The patient himself was a keen, level-headed man, advanced in years, and his symptoms were characteristic throughout. The disease ran a progressively fatal course, and death ensued.

Of the other two cases the evidence is not so complete, but in the second one the symptom-complex was a quite typical one. Of the third case there may be more doubt, but the probability is certainly in favor of hydrophobia. I do not think that in any of these three cases there is the slightest evidence that death resulted from fear or from any imaginary element whatever.

Dr. Patrick, of Chicago, tells me that he has seen both genuine and spurious cases of hydrophobia, and that there is no mistaking them. He said that a boy was once brought to him who was biting and barking and had become so dangerous that his friends had bound him, but was in an apparently hysteric condition; the attendants were in such fear that they would not release him. He sent them from the room and told the boy to stop his nonsense immediately, that he would have no more of it. The boy quieted down at once, and seemed to be as much relieved as his people were. I think it is safe to say that in every suspected case if the disease is spurious recovery will follow, but if hydrophobia is present death will surely result.

In regard to the animals mentioned by Dr. Searle, it is a well known fact that there is in the west a small skunk, smaller than our skunk, whose bite is followed by hydrophobia. It has a very objectionable habit of running around at night and biting people's toes and noses, and hunters and cow punchers are much afraid of it on this account. President Roosevelt mentioned this fact in his book on ranch life, and I have been told of it by several other persons who have lived in the west.—*Cleveland Journal of Medicine.*

### CATCHING COLD.*

Billings's Medical Dictionary defines cold "a morbid condition of the upper respiratory tract, produced by exposure to cold air or dampness; catarrh." He defines catarrh, "inflammation of the mucous membrane, especially of upper part of respiratory tract; a cold; influenza." And influenza he defines "a specific, epidemic, catarrhal fever, with inflammation of mucous membrane of upper air passages and conjunctivæ, accompanied with nervous symptoms and prostration."

The Century Dictionary defines cold "an indisposition commonly ascribed to exposure to cold; especially a catarrhal inflammation of the mucous membrane of the nose, pharynx, larynx, trachea, bronchi, or bronchial tubes. When the inflammation is confined to the air passages of the nose and connecting cavities, it is a coryza, or cold in the head. A so-called cold on the lungs is usually bronchitis, or tracheitis."

Mr. Arthur E. Durham, in Quain's Dictionary of Medicine, says: "As a predisposing and exciting cause of disease cold proves, in this country (England), year by year, more fatal in its effects probably than any other single condition or influence. Any considerable fall in the thermometer below the average standard during the colder months of the year is constantly followed by a corresponding rise in the death rate and an increase in still greater proportion in the amount and extent of sickness and suffering. . . . In the week ending December 19, 1863, in the London district, 1,291 deaths were registered. Severe frost set in, and in the week ending January 9, 1864, the number rose to 1,798. The week following, ending January 16, no fewer than 2,427 deaths were registered. This enormous increase could be attributed to no other cause than the effects of the severe cold which prevailed. The Registrar-General also shows that, after the age of from twenty to forty, the mortality from cold increases in something like a definite ratio with increasing years. General depression of the vital powers, congestion, and functional derangement of various internal organs—the lungs, liver and kidneys—catarrhal and other forms

*Read by Dudley S. Reynolds, A.M., M.D., before the Ohio Valley Medical Association at Henderson, Ky., November 11, 1901.

of inflammation of the mucous membranes, especially of the respiratory tract, but also of the intestinal canal and bladder; paralysis from central or peripheral lesion, together with rheumatism, chilblain, frostbite and gangrene constitute the list of maladies most commonly caused and fostered by exposure to the influence of cold."

Dr. Samuel Sexton, of New York, in discussing the subject of chronic catarrh of the middle ear, says: "The predisposing causes comprise all circumstances which tend to produce a permanent depression of the vitality of the body. It is a matter of common experience that changeable weather is most favorable to the development of cold, that catarrhal affections of all sorts are rife in the transition period of autumn, and still more in spring. The inhalation of bad air has been regarded as a frequent cause; this may well be doubted, however, as having the influence that is attributed to it, and wherever it does act it is associated with other causes still more powerful." He considers bad food, lack of exercise, sudden exposure, and overexertion more potent. He believes, however, excessive ventilation, by furnishing draughts of air more prejudicial, as tending to cause catarrh. Diet, he thinks, has an important bearing, especially the habit of overfeeding children. He mentions syphilis and scrofula as predisposing causes. He says: "Chronic Bright's disease and gouty and rheumatic affections predispose to catarrh, and pregnancy or chronic uterine disease may create a similar tendency."

Dr. George M. Gould, in Burnett's System, says: "One especially striking case I have reported in which, beyond doubt, the production of a severe common cold was repeatedly shown to be dependent upon ametropia." He quotes Dr. Miles as saying: "Ocular irritation was a common cause of these disorders, from the fact that, upon correcting the ametropia with suitable lenses, the usual tendency to bleed, sneeze, etc., was often entirely, or in a great degree removed, and especially when associated with asthenopia and headache."

Dr. Gould recites his experience in favorably modifying nasal affections by collyria, which, finding their way through the tear passages, favorably influences the condition of the nasal membrane.

Dr. Edw. B. Dench, of New York, says: "Naso pharyngeal catarrh is probably due to atrophic changes which take place in the pharyngeal tonsil in adult life." Eustachian catarrh, he thinks, usually arises from an acute coryza, or an acute naso pharyngitis, although it may be met with as a primary condition from exposure to cold. Occasionally it complicates light attacks of exanthemata in young adults. It may depend upon the entrance of some irritating fluid into the Eustachian tube," etc.

While Dr. E. Fletcher Ingals (in his "Treatise on Diseases of the Chest, Throat, and Nasal Cavities") is convinced that influenza is evidently caused by some powerful morbific agent in the atmosphere, he thinks nasal catarrh is most commonly caused by exposure to cold when the body is overheated; but not infrequently caused from undue heat, or the inhalation of dust, or irritating fumes or vapors. In other cases acute rhinitis is probably infectious. Among the occasional causes he mentions exposure to the rays of the sun, impetigo, or eczema, measles, scarlet fever, typhoid fever, tertiary syphilis, iodism, facial erysipelas, or extension of inflammation from the conjunctivæ, pharynx or larynx; and it is said to be caused, in some instances, by the cure of chronic discharges, such as those of otitis, ophthalmia or bleeding hemorrhoids. In writing of chronic rhinitis, Dr. Ingals says: "The disease may be induced by the frequent repetition of any of those conditions which cause an ordinary cold. It may result from inhalation of irritating substances, exposure of the throat, back, ankles, or of the whole body to cold, or from the inhalation of damp, chilly atmosphere. A predisposition to inflammation of the mucous membrane may be inherited, or acquired by frequent attacks of the acute disease. Debility and a depressed condition of the nervous system often directly favor the onset of the affection, and in many cases hyperesthesia of the terminal nerve fibers of the Schneiderian membrane is apparently the predisposing cause. In some cases it is favored by the scrofulous or dartrous diathesis." In considering atrophic rhinitis, Professor Ingals says: "The cause cannot always be ascertained, but in some persons a history of frequent colds, with more or less complete obstruction in the nares for a considerable period, dating from an exanthematous fever, and at others from an injury, leads to the belief that the affection is usually preceded by chronic catarrhal inflammation, and favors the theory that atrophy results from an antecedent hypertrophy."

Ziegler's Pathology contains the following: "In the experiments of Ponfick, Slavjansky, Ruppert and Soyka, cinnabar, coal dust, or Chinese black was introduced into the blood of animals, either directly or through the pulmonary lymphatics, by inhalation." . . . "Insoluble matters like cinnabar, coal dust, etc., remain in the tissues permanently, or are in part eliminated from the body. Experiment shows that, after many weeks, some part of the substance may still lie inclosed in cells within the tissue. During this time some movement of the substance always takes place. The carrier cells change their position, and may even enter the blood once more. In this way part of the substance is carried to exterior parts of the body, and that by various routes. In the first place, glands like the kidney, liver or mamma, whose secretions pass out of the body, may eliminate all foreign substances with the secretions. But it may also pass out through the mucous membranes, through the lungs, through wounded parts, or even through the skin. Such is especially the case when active cell migration is taking place at any of these sites. Many of the extraneous substances entering the blood are soluble and destructible, and such always disappear after a longer or shorter time. Thus, inhaled particles of chalk-dust are dissolved in the blood; fat, which circulates in great drops, rapidly disappears, and micro-organisms likewise break up and are absorbed as soon as they cease to find the vital conditions that are necessary to them." . . . "Acute nasal catarrh is spoken of as coryza, and may result from a great variety of causes, such as cold, inhalation of irritant matters, micro-organisms," etc. . . . "Chronic nasal catarrh occurs chiefly in persons who are scrofulous, phthisical or syphilitic; it is comparatively rare in persons otherwise healthy."

These extracts are from recent issues of standard text-books, and show clearly the vague conception the authors have of the meaning of terms in common use, as well as their lack of scientific methods of study.

Let us reflect that the late Professor George B. Wood's definition of catarrh has been lost sight of, viz., "Afflux of all the circulating fluids, with hyper-secretion of the glandular apparatus of the affected part." Schoenbein's experiments with ozone, in 1851, offered a scientific explanation of the often widespread epidemics of coryza. Sir Thomas Watson, in his "Lectures on the Practice of Medicine," added an extensive series of observations to strengthen Schoenbein's theory. In the course of time the few facts recorded of the reflex phenomena of disturbances of the respiratory system have been confounded with congenital defects of structure in the respiratory membrane, deformities of the nasal passages, and a varied series of local infections.

All the irritations of the nose and pharynx, all the structural defects, and all the local infections are superstitiously attributed to cold. Even the reflex disturbances of the upper respiratory system, from errors of refraction in the eyes, from ovarian and uterine disease, and from bleeding hemorrhoids, are said to cause "common cold." If we accept such statements we must believe that hereditary defects of the Schneiderian membrane, deformities of the nasal passages, reflex irritations, infectious inflammations, and irritation from inhaled foreign insoluble matter, all produce catarrh, and that catarrh means common, or bad cold.

A little attention to the microscopic examination of nasal mucus and the sputum will quickly disclose the presence of any foreign matter or living organisms. Ocular inspection of the affected membrane, under artificial illumination, will at once disclose to the practiced observer the presence of structural changes, malformation of the passages, or the presence of any inflammatory products. People who believe in the supernatural in medicine think every affection of the respiratory tract is either an inherited or acquired bad cold, and every affection of the nasal membrane catarrh, even though the surface be perfectly dry.

Now, if catarrh means anything, it means to flow down, a liquid discharge. Since all infectious inflammations of the mucous membrane discharge some sort of fluid, they must all be catarrhal. Catarrh, then, may mean, in fact, any sort of discharging surface. There can be no more dry catarrhs than dry rivers. Cold necessarily means low temperature, absence of heat. In medicine, however, it means superstition, and the worst sort of ignorance. Careless medical writers are in the main responsible for the ignorance of the lay readers in all such matters.

Let us teach the people to understand that the injurious properties of draughts of air lie in the foreign matter they contain rather than the depression of body temperature produced by them.

Clear air is more wholesome and invigorating when cold than when warm; foul air is equally hurtful, whether cold or warm. The reason cold weather is attended with the more widespread prevalence of respiratory disease than warm weather is easily understood if we consider for a moment the constantly increasing variety and quantity of foreign matter in the atmosphere of closed apartments. In cold weather, people going in and out bring into the hallways all kinds of matter found on the pavements and in the pathways, and those who remain indoors are breathing all the time such matters; hence, it is easily seen why women and children are the most constant sufferers, excepting alone those men who work about stables. The stable boys are nearly all afflicted with some sort of infectious disease of the upper air passages. Horses wallow in dirt, often containing excrementitious matters in a state of moisture; the hairs rubbed in this filth are soon dried, and in the morning the stable boy comes with his curry-comb and brush, and fills the air about him with fine particles of it. This matter frequently contains infectious germs. These boys, walking about the stables, go into the house presently, carrying a quantity of dirt into the hallways, and often into the rooms.

Now, as age advances mortality must necessarily increase. Confinement indoors in vitiated atmosphere means almost necessarily filling the air-passages with insoluble matter, and likewise with some form of infection, most commonly of the staphylococcus variety. Add these sources of disease and the wonder is that anybody escapes.

It is easy to understand why catarrhal inflammations of an infectious character are "rife in the transition period of autumn, and vastly more prevalent in early spring."

There can surely be no kinship between the reflex irritation that provokes sneezing and spasmodic cough, with more or less coryza, and a staphylococcus infection, or any other sort of infection. Infections are always due to the presence of specific ferments, ptomaines or other poisons. It would be just as impossible for the sudden chilling of the surface of the body, which brings on an attack of sneezing and temporary coryza, to generate an infectious inflammation as it would be to produce horses and cattle by mere changes of temperature; for the good reason that infectious inflammations of the mucous membrane cannot arise without the presence upon them of the spores of some one of the micro-organisms which grow in mucus.

I have often suffered from violent fits of sneezing from sudden chilling of some part of the body, and for years I kept plate glass at hand for the purpose of collecting the expelled matter; and it may be important to remark just here that sneezing through the nose inevitably excoriates portions of its lining, besides causing great swelling and closure of the passages. I therefore throw the mouth wide open when I am going to sneeze, and by sneezing on a piece of plate glass, about twelve inches square, it is easy to collect the most tenacious portion of the mucoid matter expelled. Subjecting these portions to microscopic examination immediately after the sneezing has ceased, I have found small portions of wool fibre, hair, cotton, straw, wood, and an infinite variety of other foreign substances.

Those of us who have seen in the slanting rays of the sun the myriads of glittering particles of foreign matter whirling in the air, of even well-ventilated apartments, can have no difficulty in comprehending the mischief which must result from inhaling such matters.—*Lancet Clinic.*

---

### THE TREATMENT OF SYPHILIS, WITH SPECIAL REFERENCE TO THE BEST METHODS OF ADMINISTERING MERCURY.*

The author calls to mind the facts that mercury has been used in the treatment of syphilis for over 400 years, and there are few physicians to-day who do not use it in some form. Although

*Abstract of an original paper, by Winfield Ayres, M.D., Genito-Urinary Surgeon, Bellevue Hospital, O. D. P., New York; Instructor in Genito-Urinary Diseases in New York University and Bellevue Hospital Medical College; Instructor in Genito-Urinary Diseases in the New York Post-Graduate Hospital, etc., in *The Lancet* (London, Eng.), October 19, 1901.

the method of treatment with mercury is still discussed, he is firmly of the opinion that there is no hope of eradicating the disease unless the full dose is given constantly for something like three years. The treatment should begin just as soon as the diagnosis can be made. There is no ground for supposing that enucleation of the chancre has the effect of aborting the disease. If a positive diagnosis cannot be made from the appearance of the initial lesion, general tonic treatment should be instituted.

In some cases the protiodide controls the symptoms, but in the majority it is of very little use. Experiments with Mercurol were conducted at Bellevue Hospital, for eight and a half months, with 180 cases; the histories of 95 of these are recorded. The remainder could not be kept under observation and are therefore passed over. The dosage of the Mercurol, regulated either by reaching the point of tolerance or control of the disease, varied from one half to six grains. In 64 of the 95 cases the disease was controlled as follows: in two weeks, 8; three weeks, 12; four weeks, 14; five weeks, 6; six weeks, 5; seven weeks, 2; two months, 8; ten weeks, 2; three months, 5; and four months, 1. The remainder are marked thus: decidedly improved, 17; improved, 8; no improvement in two weeks, 3; no improvement in four weeks, 1; and no improvement in three months, 2. The latter were all dispensary patients and it is uncertain whether they took their medicine regularly.

The writer states that his plan was to increase the dose steadily from one grain until the symptoms were controlled, or until a slight tendency on the part of the teeth and gums to become tender. If the symptoms were not controlled before the physiological effect of the Mercurol made itself felt, small doses of potassium iodide were added, and in every case where the Mercurol was taken according to directions, with the exceptions noted above, the symptoms were controlled.

In 67 out of the 95 cases tabulated, no other medicine than Mercurol was given. In 15 out of the remaining 28, the addition of iodide of potassium was found to be sufficient to control the disease, while in 6 others the addition of an iron tonic sufficed for this purpose.

The cases are not reported at length, but a few of the more remarkable results and some cases in which other medicines failed to control the disease are briefly mentioned.

Case 1 had been taking bichloride for one month with very little improvement. Under Mercurol, three grains maximum dosage, the symptoms were under control in five weeks.

Case 2 had been under biniodide of mercury (one-sixteenth of a grain) and potassium iodide (five grains), which caused iodism. His symptoms were controlled in one month under half a grain of Mercurol.

In case 3 unguentum hydrargyri had failed to control the disease. The patient was put on Mercurol and the dosage pushed up to six grains three times a day. The disease was thoroughly under control in seven weeks.

Case 4 had been on three-eighths of a grain of biniodide of mercury and twenty grains of potassium iodide for two months. The medicine caused nausea and vomiting. Having been put on Mercurol and the dosage gradually increased to five grains three times a day, the symptoms were controlled in three weeks.

Case 5 had been taking hydrargyrum bichloride (one-twelfth of a grain) three times a day, under which an eruption on his face had faded, but the eruption on his body still persisted. His symptoms disappeared in two weeks under a maximum dose of three grains of Mercurol three times a day.

Case 6 had been on bichloride of mercury (three sixteenths of a a grain) for three months, in spite of which he had palmar syphilide of an eczematous variety. All appearances of the disease disappeared after he had been one month on Mercurol, his maximum dose being three grains three times a day.

Case 7 had been taking one-quarter of a grain of Mercurol and fifteen grains of potassium iodide, with the result that the eruption had decidedly improved, though not to the extent that it should have done. There were thickened red patches on the face, covered with scaly eruptions. The symptoms almost entirely disappeared within three weeks under a maximum dosage of five grains of Mercurol three times a day and fifteen grains of potassium iodide.

Case 8 had been treated with inunctions of mercury, under which the eruptions disappeared, but the pains in the bones still persisted. He was relieved in three weeks under a maximum dosage of four grains of Mercurol three times a day.

Case 9 had been taking other forms of mercury for six months. The form which had done him most good was bichloride. Yet one-fifth of a grain did not entirely control the disease. He had been taking that for two months when he was placed on Mercurol. The dosage in his case was pushed up to six grains three times a day, and at the end of seven weeks all his symptoms had disappeared.

Case 10 had been taking medicine off and on for two years, but his symptoms never disappeared entirely. After being two weeks on Mercurol (two grains three times a day) with the addition of potassium iodide, all symptoms had disappeared.

Ayres, in conclusion, states that he uses Mercurol in his private practice to the exclusion of all other drugs. His experience is that he gets better results. He has found no form in which mercury can be given with such good results as that of Mercurol.

---

### THE TREATMENT OF NASAL CATARRH BY THE GENERAL PRACTITIONER.*

I have long entertained the view that the general medical practitioner neglects to treat his patients for catarrh and sends them to a specialist when he could successfully manage these himself. In fact, the treatment of catarrh is very simple and the results which follow correct and systematic treatment are very satisfactory. In practice, two forms of chronic nasal catarrh are met. These are hypertrophic rhinitis and atrophic rhinitis.

The hypertrophic form is more generally seen, and is characterized by a thick mucous discharge from the nose, great liability to colds, obstruction of one or both nostrils, which forces the patient to breathe through his mouth, nasal intonation of the voice. There is more or less headache and the sense of smell is lost or impaired. There is dryness of the throat, deafness and other symptoms showing the extension of the disease to neighboring organs. Exostosis of the osseous structures often is seen.

Atrophic rhinitis (ozena) is characterized by a sense of dryness in the nose and throat, a thick, purulent discharge and the expulsion of discolored crusts and an offensive putrid odor. The sense of smell is impaired and the patient is weak and anemic.

The mucous membrane is dry and glazed, but in advanced cases ulceration and necrosis are present.

The treatment consists of applications directly to the diseased area and the administration of such internal remedies as will correct any coexisting disease or morbid state. In some cases where there is occlusion by exostosis the resources of surgery must be invoked.

*By Eugene C. Underwood, M.D., Surgeon B. & O. S. W. R. R.; Surgeon K. & I. B. Co., etc., Louisville, Ky. Abstract from St. Louis Medical and Surgical Journal, July, 1901.

Let me examine more in detail the treatment of the types of nasal catarrh.

In simple chronic hypertrophic rhinitis the results of treatment will be most flattering. In a case attended with no constitutional disease nothing is necessary beyond having the patient spray the nasal mucous surface with a solution composed of equal parts of water and Hydrozone every three hours.

If the case has persisted some time and the patient has an amount of mucous discharge, I have him take twenty drops of balsam of copaiba four times daily. The Hydrozone is not only a disinfectant and germicide, but its curative action on the inflamed mucous membranes is speedy and is not equaled by any other drug I have ever used. When the patient is anemic I have him take iron, and any other drug is used when it is called for by any associated disease or morbid condition, but the Hydrozone spray is used in all cases.

In the atrophic variety we shall have to use the same local application. The Hydrozone at once overcomes the offensive odor and takes off the purulent crusts.

These crusts must be treated with cod liver oil, iron and such other remedies as will bring up the general health.

Here are a few clinical histories:

Mr. R. H. M., age 60, had been a sufferer for two years. There was no exostosis, but when he had a cold he could breathe only through his mouth. He was in good general health, so I had him buy an atomizer and use a spray composed of equal parts of distilled water and Hydrozone. He sprayed the mucous surface of the nose every three hours. On this he made rapid improvement and in three weeks had no further symptoms.

S. M. T., age 18, had chronic hypertrophic nasal catarrh in which the mucous discharge was very abundant, and this was associated with dryness of the throat and constant desire to hawk and spit. She used the Hydrozone and water spray, and took fifteen drops of balsam copaiba three times daily. I had the pleasure of seeing this young woman go along to complete recovery in a period of six weeks.

Mrs. R. J. C., age 49. This lady had atrophic rhinitis and as soon as she came near you the putrid odor asserted itself. Her general health was lowered. I had her use the Hydrozone and water spray and take cod liver oil internally. She spent last winter in Cuba, and has just gotten home greatly improved in general health and her catarrhal disease is better.

She says the spray effectually destroys the disgusting odor and that scarcely any discharge now appears.

I expect to see this patient entirely well in several months.

### A REMEDY PROPOSED FOR THE EVIL OF SUBSTITUTION.

BY J. D. WILLIAMS, M.D., NEW YORK.

There can be no subject of more importance to physicians than the violation of their confidence on the part of a dishonest dispensing druggist. Law will not make a dishonest man honest, but the right law properly executed will prevent a criminal's further infliction of injury upon society. The requirement of a license to all druggists who dispense drugs or medicines, revokable upon the licensee's being convicted of substituting any ingredient drug or medicine other than, and in lieu or instead of, that specified in the prescription, order or request in writing, of any physician, would go a long way to aid in the matter of honestly filling prescriptions. Let the medical societies induce their respective State Legislatures to enact a law requiring such a license, with a simple and practical procedure for establishing the guilt and enforcing the penalty against infraction, and the practice of substitution would soon cease.

Let proceedings for revocation of license be before the court, board, or officer empowered to issue the license, and be set in motion at the relation of either the Board of Health, a local medical society, or the purchaser upon whom the fraud and imposition had been done, or of the physician by whom the prescription or order was issued or given, or of any person, firm or corporation for whose brand or make of drug or medicine the substitution had been perpetrated. Let the licensing board, court, or officer be empowered to issue citations, subpœnas for witnesses, to administer oaths, and be given all other requisite powers for duly trying the issues and revoking the license of the guilty.—*Exchange.*

---

### A HANDSOME GROUP OF PORTRAITS.

About a year ago, within the short period of three weeks, four eminent and distinguished physicians departed this life. Two of these well known men (Hunter McGuire and Lewis A. Sayre) were surgeons, and two (Jacob M. Dacosta and Alfred L. Stillé) physicians, and three of them were ex-Presidents of the American Medical Association. Believing that the rank and file of the profession would appreciate and preserve portraits of these representative practitioners and teachers, the Arlington Chemical Company commissioned a competent artist to paint them in oil in the shape of a panel suitable for framing, and have reproduced the painting for distribution to their friends in the profession. This handsome and thoroughly worthy group of portraits is now being mailed, and if any physician is, perchance, omitted from the list, a request will bring a copy.

